# Does Bird Diversity Affect Public Mental Health in Urban Mountain Parks?—A Case Study in Fuzhou City, China

**DOI:** 10.3390/ijerph19127029

**Published:** 2022-06-08

**Authors:** Weizhen Xu, Dulai Zheng, Peilin Huang, Jiao Yu, Ziru Chen, Zhipeng Zhu, Jianwen Dong, Weicong Fu

**Affiliations:** 1 College of Landscape Architecture, Fujian Agriculture and Forestry University, 15 Shangxiadian Rd, Fuzhou 350002, China; 1201775052@fafu.edu.cn (W.X.); 1201775063@fafu.edu.cn (D.Z.); 3201726032@fafu.edu.cn (P.H.); 3201726093@fafu.edu.cn (J.Y.); fjdjw@fafu.edu.cn (J.D.); 2 College of Architecture and Urban Planning, Fujian University of Technology, 33 Xuefunan Rd, Fuzhou 350118, China; ziru.chen@alumni.ubc.ca (Z.C.); 19912151@fjut.edu.cn (Z.Z.); 3 Engineering Research Center for Forest Park of National Forestry and Grassland Administration, Fuzhou 350002, China

**Keywords:** mental health, urban mountain parks, bird diversity, urban planning

## Abstract

Intensified urbanization has caused a linear decline in the quality of urban biodiversity and indirectly harms our current human settlement environment. Urban mountain parks provide a refuge for the animals and plants and play a vital role in satisfying residents’ lives. At present, few studies are focusing on the impact of biodiversity on human mental health benefits of urban mountain parks in high-density construction areas along the coast of the Eastern Hemisphere. Here, we examined the relationship between bird abundance, Shannon diversity, Simpson diversity, and Richness and momentary mental health (positive, negative, and anxiety) in urban mountain parks. The timed species counts method was used to conduct three surveys of birds in urban mountain parks, and linear regression was performed on the relationship between bird diversity and mental health among sites. According to the regression model results, we found no significant correlation in any disturbance levels. As urban mountain parks are an essential part of the human settlement environment, how to improve the biodiversity and mental health of urban mountain parks is one of the focuses of research on biodiversity well-being in the future. Urban planning authorities and public mental health researchers should pay attention to the importance of biodiversity in urban development and consider how to realize the beautiful vision of the harmonious coexistence of humans, animals, plants, and the environment in which we live.

## 1. Introduction

In China, about 60% of the population lives in urban areas now, as urbanization continues in other parts of the world. Rapid urbanization has reduced the coverage of green space in urban areas [1], resulting in reduced opportunities for urban residents to contact nature [2,3]. Previous studies have shown that mental health problems (such as anxiety and depression) are much more common in urban areas than in rural areas, which may be detrimental to human well-being to some extent [4]. Urbanization also puts tremendous pressure on regional biodiversity and urban ecosystem function [5]. However, urban green spaces (e.g., parks, gardens) provide refuge for some plants, animals, and other species and a virtual environment for the public to enjoy physical activity, nature, and social interaction, promoting public health and well-being. Therefore, urban green space planning, design, and management are vital to biodiversity conservation, ecosystem service enhancement, and the promotion of human well-being [6].

Emerging literature also emphasizes the importance of urban parks and green spaces for public health [7,8,9]. Additionally, it has been stated that the health benefits of green space depend on its quality [10,11]. Studies about the relationship between urban parks and public mental health in China are generally as follows: (1) urban parks can improve the cognitive function of recreational visitors, provide a restorative experience, reduce mental stress, and promote recovery from attention fatigue [12,13,14,15], or (2) recreational activities in urban parks and green spaces can promote public place attachment [16] and other positive benefits [17,18,19,20]. Similarly, experimental evidence showed that people prefer to look at multi-dimensional scenes with more biodiversity rather than just green spaces and believe they are more refreshing [21,22,23]. So far, researchers have studied various psychological outcome indicators (such as psychological distress, the incidence of mild mental illness, etc.). Still, mental health is rarely used as a research focus [24]. Some researchers propose to improve the connection between mental health and green spaces [25,26]. Others believe that human beings are “biophilic“ [27,28], and they relate to nature through the continuous evolution of genes adapted to the natural environment, which leads to positive or adverse reactions to specific stimuli reaction (such as running water, sharp-mouthed species). However, past evidence can support the argument that experiencing more diverse biodiversity or green space will bring a stronger sense of happiness [29,30].

At present, studies have demonstrated that there is indeed a contribution of biodiversity to well-being [29,31,32,33]. Luck et al. (2011) found that public well-being is positively associated with a range of natural characteristics, including species richness and abundance of birds [34]. A study from Europe also confirmed that bird diversity has a strong positive impact on public life satisfaction [31]. To further clarify the impact of biodiversity space on public mental health, it is necessary to understand which characteristics can enhance or weaken general well-being [31]. Specifically, for decision makers tasked with improving the quality of human settlements and the environmental quality of species, it is of greater significance to distinguish the role of biodiversity in the relationship between humans and nature. There are also significant geographic gaps in the experimental space carried out on the relationship between biodiversity and well-being and few studies from the Eastern Hemisphere, especially China. Because China’s urbanization process has more distinctive characteristics than other countries, it is more representative to choose China as the research object [35].

Here, we explored the relationship between bird diversity and public mental health in urban mountain parks in Fuzhou. Birds were selected as the object of this study mainly because of the low cost of bird monitoring. Additionally, they can be used as indicator species for ecosystem service functions [36,37]. In addition, urban mountain parks are generally covered with high-density vegetation and suitable habitat quality [38]. They have become essential refuges and habitats for birds, and different levels of bird diversity have been found in various anthropogenic disturbances of urban mountain parks. Then, we hypothesized that this observed change in bird diversity along different anthropogenic disturbances would be related to changes in public mental health (positive, negative, and anxiety). This study linked biodiversity to the well-being of mountain parks and addressed essential knowledge gaps.

The questions we asked were:

1. Are there any significant changes in bird diversity that vary along a different gradient of urban mountain parks? What are the main factors affecting its changes?
2. Are there differences in momentary mental health under a different gradient of urban mountain parks?
3. Does bird diversity affect momentary mental health under different anthropogenic disturbances?

## 2. Materials and Methods

### 2.1. Study Area

Fuzhou is in southeastern China and is the capital of Fujian Province. It has a subtropical humid monsoon climate with long summers and short winters. According to statistics from the Fuzhou Municipal Bureau of Statistics, at the end of 2020, the permanent population of Fuzhou was 8.32 million, and about 70% of the population lived in high-density urban areas. Fuzhou City has 58 mountains of different sizes within its jurisdiction as a mountainous city. The Fuzhou Municipal Government has developed more urban mountain parks to meet residents and foreign tourists.

Due to the particularity of the topography, history, culture, and social culture of mountain parks, decision makers are faced with more severe planning, maintenance, management, and challenges [39]. Regardless of the country, in densely populated urban living environments, green space and biodiversity are more important and needed for urban residents. Taking typical mountain parks in Fuzhou as an example, this study aims to analyze the impact of bird diversity on public mental health in urban mountain parks with different anthropogenic disturbance gradients.

**Figure 1:**
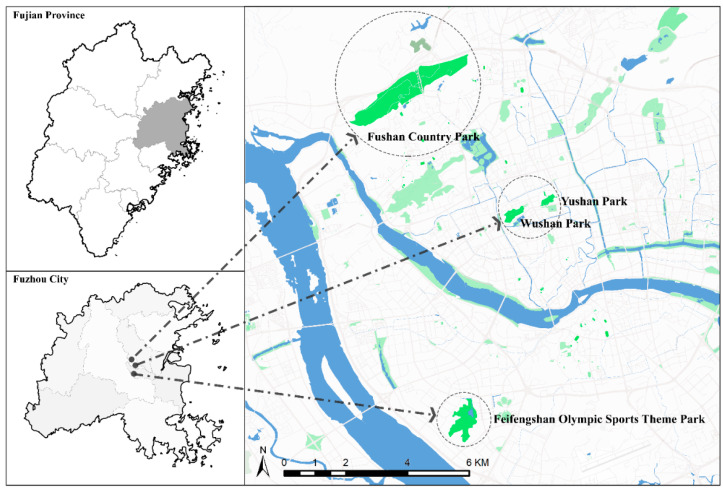
Map of the study area shows the location of the study sites adapted from Google Earth.

To ensure the representativeness of data acquisition, we selected the urban mountain parks in the main urban development areas and districts, including Gulou District, Cangshan District and Taijiang District [40,41,42]. According to the urban planning and vegetation conditions of Fuzhou, the urban mountain parks are screened according to the following requirements: (1) lie in different locations and be representative; (2) have diverse plant composition, typical landscape habitats, and abundant biodiversity resources [42]. Finally, four urban mountain parks were chosen, Fushan Country Park, Feifengshan Olympic Sports Theme Park, Wushan Park, and Yushan Park, respectively. The above urban mountain parks are in three areas with different construction densities in Fuzhou, with high accessibility, rich vegetation, and bird resources, and represent the urban mountain parks of Fuzhou (see Figure 1).

#### 2.1.1. Classification Standard for Anthropogenic Disturbance Gradient

The classification of anthropogenic disturbance gradient was based on four criteria:

Firstly, parks need to be in a formal protection status under the management of the state administrative organs. In this regard, urban mountain parks with the lowest proper protection were classified as having a high disturbance level. This is based on the logic of protecting and minimizing the impact of urbanization and artificial transformation [43]. Secondly, the relative amount of people allowed to visit or enter. Having more visitors or easy entering means a more significant negative impact on the site and, therefore, a higher interference score. Third, the number of entrances that the visitors and vehicles can pass through. The more entrance there are, the higher the disturbance level. Fourth is the relative openness of the site in terms of vegetation coverage. The most open site means higher human disturbance and, therefore, the highest disturbance level by applying rough supervision classification to Landsat 8 images of the 90 m digital elevation model. Using satellite map estimates the classification and evaluates vegetation coverage in ArcMap v10.2, divided into three categories: gray space, blue space, and green space.

#### 2.1.2. Three Levels of Anthropogenic Disturbance Gradient

**Figure 2:**
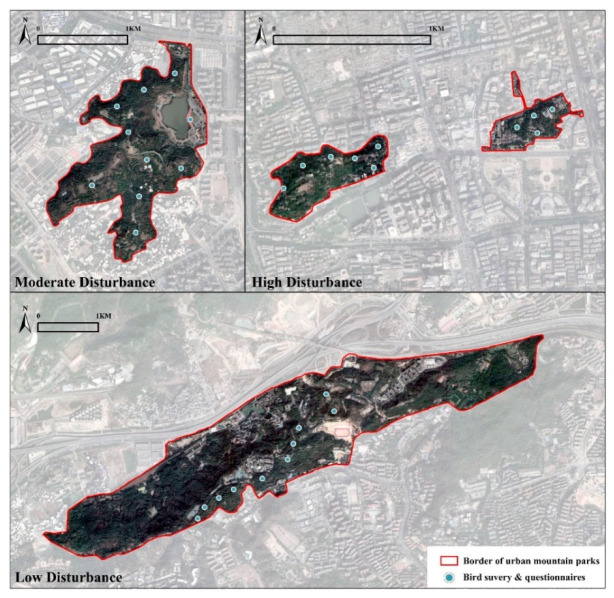
Sites in three disturbance levels (low disturbance, *n* = 10; moderate disturbance; *n* = 10; high disturbance, *n* = 10) were used for bird surveys and questionnaires.

According to the above four criteria, Yushan Park and Wushan Park are in the city center, and the two parks have the largest number of people and entrances and do not charge any fees. Feifengshan Olympic Sports Theme Park is located between Fuzhou’s third and second ring roads. The flow of people is lower than that of Wushan Park and Yushan Park. There are open squares at the entrances, and these allow visitors to move freely. Fushan Country Park is the furthest away from the city center, but it is covered by virgin forests and relatively high green space. Additionally, it has a low proportion of gray space, such as square paving and pathways. The flow of visits only increases during holidays, but overall, it has weak reachability. Therefore, we classified Fushan Country Park and Feifengshan Olympic Sports Theme Park as low-disturbance and moderate-disturbance parks, respectively. In contrast, Wushan Park and Yushan Park are classified as high-disturbance parks (see Figure 2).

### 2.2. Questionnaire Design and Delivery

We collected data from questionnaire surveys and bird surveys in the three anthropogenic disturbance urban mountain parks. Firstly, we randomly selected 10 sample sites in sites of each level, with low disturbance (Fushan Country Park, *n* = 10), moderate disturbance (Feifengshan Olympic Sports Theme Park, *n* = 10), and high disturbance (Wushan Park, *n* = 6, Yushan Park, *n* = 4); the distance between sample sites needed to be at least 100 m to ensure space independence. Since it is necessary to match the bird diversity at the sample level with the momentary mental health, the questionnaire needed to be consistent with the bird survey scope. To ensure the validity and consistency of the questionnaire, the number of questionnaires between the sites was controlled within ten copies, and the minimum age of interviewees was 18 years old.

We invited participants to answer a questionnaire about “feelings in urban mountain parks.” Additionally, the respondents were told they are participating in a study on “whether bird diversity in urban mountain parks impacted public mental health”. Before the experiment began, we asked respondents to observe the environment around the sample sites for 1–3 min, focusing on the distribution of bird species, which is done to reduce the influence of other environmental variables on the respondents [9,23,26]. The first two questions were used to explore the basic information of urban mountain park visitors, including their frequency of visiting mountain parks (visiting frequency) and the type of people with whom they visited. The above question was first raised to reduce response deviation [31]. For measuring the visit frequency, we asked: “How often do you visit mountain parks?”, five answer options (daily, weekly, monthly, yearly, more than one year), and “who are you coming with today?”, five answer options (child, friend, parent, alone, and others) to record the type of fellows. Then, we asked them “how familiar with bird species in Fuzhou” on a three-point scale (1 = completely unfamiliar, 2 = slightly familiar, 3 = familiar).

Momentary mental health was considered by three emotions: positive, negative, and anxiety, using effective scales commonly used in nature well-being research [33]. We asked the interviewees to estimate how they feel at the moment. Additionally, the interviewee was explicitly asked only to consider the range within a radius of 50 m, which corresponded to the bird survey area. The Positive and Negative Affect Schedule (PANAS) was used to test the respondent’s ten positive and ten negative emotions on a 5-point scale (1 = not at all, 2 = slightly, 3 = moderately, 4 = quite a bit, 5 = extremely) [44]. Each group of 10 emotional scores was added to form a continuous (10–50 points) measure of positive and negative influence (Table 1(a)). The six-item State-Trait Anxiety Inventory (STAI) [45] uses the same questions as PANAS to measure anxiety (Table 1(b)). To be consistent with PANAS, we changed the answer options from the original four points to five points to reduce the confusion of some interviewees. We negatively scored the negative sentiment indicators in STAI, then added up all the scores and multiplied them by 3.33 to obtain a total score of 20–100. Cronbach’s α function in SPSS 23.0 was used to check the internal consistency of each scale [46].

Combined with the census data from the Fuzhou Municipal Bureau of Statistics in 2020, we collected demographic data about Fuzhou to determine whether all the questionnaire samples we collected represented the population of Fuzhou. Cards were used to display question options to reduce the probability of respondents skipping the question. In return, each participant was given a postcard as a gift for taking part in our study. The questionnaire was delivered from 10 November to 20 December 2021 and was only carried out in sunny and windless weather (08:30–17:30).

### 2.3. Bird Sampling

We used the Timed Species Counts (T.S.C.) technique after Bibby et al. (2000) [47] with equal effort in each site. Each sample site was counted three times, and the Chao index [48] was used to check whether the bird survey data were saturated. The survey time was selected with clear weather without wind, and it was conducted three hours after sunrise and three hours before sunset. Each survey lasted 15 min. All birds were seen and heard within 50 m of the sampling spot counting center, including those recorded flying no more than 25 m above the survey area.

The surveys were conducted between October and December 2021. This spanned the middle of the wet to the start of the dry season, and therefore, we accounted primarily for bird residents and breeding in Fuzhou. Since the three different anthropogenic disturbance sites were several kilometers away from each other, inter-site bird movements were unlikely to happen. Therefore, there was no possibility of spatial autocorrelation of α-diversities across the three disturbance zones [49]. Furthermore, within sites, the survey points were placed in such locations to eliminate the possibility of internal bird movements. Both these constraints helped in maximizing sampling independence.

### 2.4. Statistical Analyses

Firstly, in order to clarify the distribution of bird diversity in urban mountain parks in Fuzhou, we selected four diversity indices: richness, abundance, Shannon–Wiener diversity, and Simpson diversity, which are also frequently used in the field of biodiversity [43]. The number of bird species per disturbance level was the species richness, while the abundance was the number of individuals per disturbance level. Shannon–Weiner diversity index is an index used to describe the disorder and uncertainty in the occurrence of individuals in bird species. The higher the uncertainty, the higher the diversity.

Shannon–Weiner diversity index calculation formula is:

*H*′ = −Σ*P_i_lnP_i_*

*P_i_* is the ratio of the number of individuals of bird species *i* to the total number of individuals in the community, *i* = 1, 2, ..., S. S is the total number of bird species.

The Simpson diversity index is the probability that the number of individuals obtained from two consecutive samples of bird community belong to the same species.

The Simpson diversity index calculation formula is:

$$D\;=\;1\;-\Sigma P_{i}^{2}$$

*P_i_* is the ratio of the number of individuals of bird species *i* to the total number of individuals in the community, *i* = 1, 2, ..., S. S is the total number of bird species.

The above indices are all calculated in the “vegan” package in R [50]. Secondly, to compare whether there are differences in bird diversity and public mental health between different sites, we used Wilcoxon rank-sum test (non-parametric statistical hypothesis test) [51] and chi-square test [52], both of which were calculated by the “wilcox.test” function and the “chisq.test” function, respectively, and both tests are applicable to datasets with less than 50 samples. Thirdly, for comparing the composition of bird communities in urban mountain parks under different disturbance intensities, we used non-metric multi-dimensional scaling analysis (NMDS), which was performed in the “metaMDS” function. NMDS analysis could simplify samples or variables in multi-dimensional space to low-dimensional space for positioning, analysis, and classification while retaining the original relationship between objects, reflecting the order relationship between objects. Statistical differences were then quantified using Analysis of Similarity (ANOSIM), calculated by the “anosim” function in R. Finally, for assessing whether levels of biodiversity in urban mountain parks under different disturbance intensities can predict well-being, we performed a general linear model, and ‘site’ was set as a random effect to control for independence, which was performed in the “lme4” package [53]. The predictor variables in the regression model were bird diversity (richness, abundance, Shannon–Wiener diversity, and Simpson diversity), and the response variable was public momentary mental health. Next, we focus on the numerical variable of sentiment and variance inflation factor (VIF) was used to check for multicollinearity and avoid outliers in the model [54]. Usually, when VIF < 10, there is no multicollinearity in the model; when 10 ≤ VIF < 100, there is strong multicollinearity; when VIF ≥ 100, there is particularly serious multicollinearity. Then, we examined model fit, overdispersion, and homoscedasticity. All our statistical analyses were performed in R version 3.5.0 [55].

## 3. Results

### 3.1. Momentary Mental Health Varies along the Disturbance Gradient

In the 30 sites where we distributed the questionnaires, we collected 603 questionnaires, excluding 10 invalid questionnaires, for a total of 593 valid questionnaires (98.32% effective). The number of valuable questionnaires collected in mountain parks of different levels was 193 for low disturbance, 195 for moderate disturbance, and 205 for high disturbance, respectively. In all the questionnaires, males accounted for 53.46%, females accounted for 46.54%, and most were aged between 18 and 55. Combined with the population data of the Fuzhou Municipal Bureau of Statistics, according to the (China) Fujian Province’s seventh national census bulletin, the permanent population of Fuzhou is about 8.29 million, with males accounting for 51.28% and females accounting for 48.72%, indicating that the ratio of men to women in the interviewees was representative of Fuzhou.

There were significant differences in the age groups visiting mountain parks of different levels (X^2^ = 33.089, df = 8, *p* = 0.000). Among them, the proportion of 18 to 24 years old was the least among the three gradients, lower than 5% of the number of visitors. Among our respondents, people over 55 years old counted the highest proportion, and the proportion was ranked as high disturbance (58.05%) > moderate disturbance (51.79%) > low disturbance (49.74%). The visit frequency of the different disturbance level mountain parks was significantly different (X^2^ = 70.374, df = 8, *p* = 0.000). The monthly visit frequency of the low disturbance (37.31%) was higher than that of the high disturbance (12%), but the weekly visit frequency of high disturbance (58.05%) was higher than that of low disturbance (26.94%). There is also a statistically significant difference in “visit frequency” (X^2^ = 92.878, df = 8, *p* = 0.000). Additionally, at the moderate disturbance, the largest number of people visited with friends (47.18%). “Alone” counted the most significant proportion among the three gradients, and the ranking was high disturbance (57.15%) > low disturbance (29.01%) > moderate disturbance (18.97%). The majority of respondents (88.36%) were either “completely unfamiliar (30.02%)” or “slightly familiar (58.34)” with bird species in Fuzhou. Those respondents who were “familiar” with bird species in Fuzhou (11.74%) were generally birdwatchers or researchers in biodiversity-related fields.

**Figure 3:**
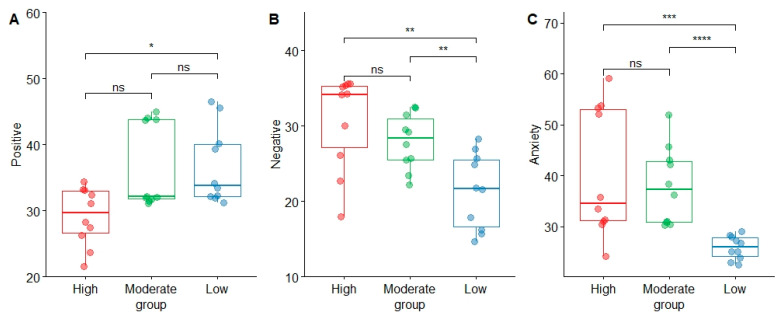
Differences in visitors’ positive (**A**), negative (**B**), and anxiety (**C**) emotions vary along three disturbances of urban mountain parks in Fuzhou, China. Boxplots show a range of data about the median (bold horizontal line), with the colored box depicting the 25th and 75th quartiles. Statistical significance level of analysis with Wilcoxon rank-sum tests (ns = not significant; * = *p* < 0.05; ** = *p* < 0.01; *** = *p* < 0.001; **** = *p* < 0.0001).

All three scales measuring momentary mental health showed good internal consistency (Cronbach’s α: positive affect = 0.956; negative affect = 0.944; anxiety = 0.888). It can be seen from Figure 3A that both low disturbance and moderate disturbance had higher scores, and there was a significant difference between low and high disturbance (*p* = 0.015), but no significant difference was found in the remaining pairwise comparisons. Among the negative emotions, the low disturbance had a lower emotional score. It is significantly different from the moderate and high disturbance, with *p*-values of 0.000 and 0.001, respectively (see Figure 3B). Additionally, we also found the same pattern in anxiety. Low grades had lower anxiety, and there is a statistically significant difference between moderate and high disturbance levels (see Figure 3C).

### 3.2. Bird Diversity Changes along the Disturbance Gradient

**Figure 4:**
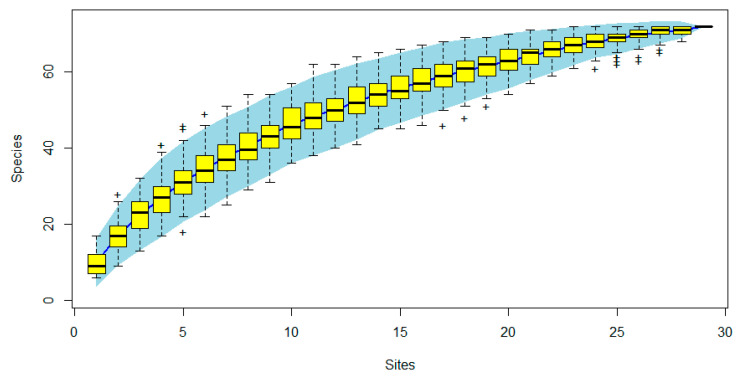
Estimated bird species richness rarefaction curve from 30 sites sampled in three disturbance levels urban mountain parks in Fuzhou, China. The colored area indicates 95% confidence intervals. “+, ‡” stands for an outlier of the box plot. The curve tends toward asymptote, indicating sufficient sampling effort in the whole site.

We observed 1524 birds, and 25 species, 41 species, and 59 species were found in high, moderate, and low disturbance, respectively. The species accumulation curve showed that bird species tend to be saturated as the number of sampling locations increases, indicating no or few unrecorded birds (see Figure 4).

Among the disturbance gradients, the top three species with the largest number, ranked high to low, are Japanese White-eye, Chinese Bulbul, and Tree Sparrow. Japanese White-eye occupied a dominant position in the three disturbance levels (high disturbance, 39.21%, moderate disturbance, 22.22%, and low disturbance, 16.88%). Chinese Bulbul was followed by high disturbance = 9.31%, moderate disturbance = 13.63% and low disturbance = 9.14%. Interestingly, we found no Tree Sparrow in the low disturbance, because the Tree Sparrow likes to live in areas with strong human activities. Still, the low disturbance had less affected by human interference, which may be less attractive.

**Figure 5:**
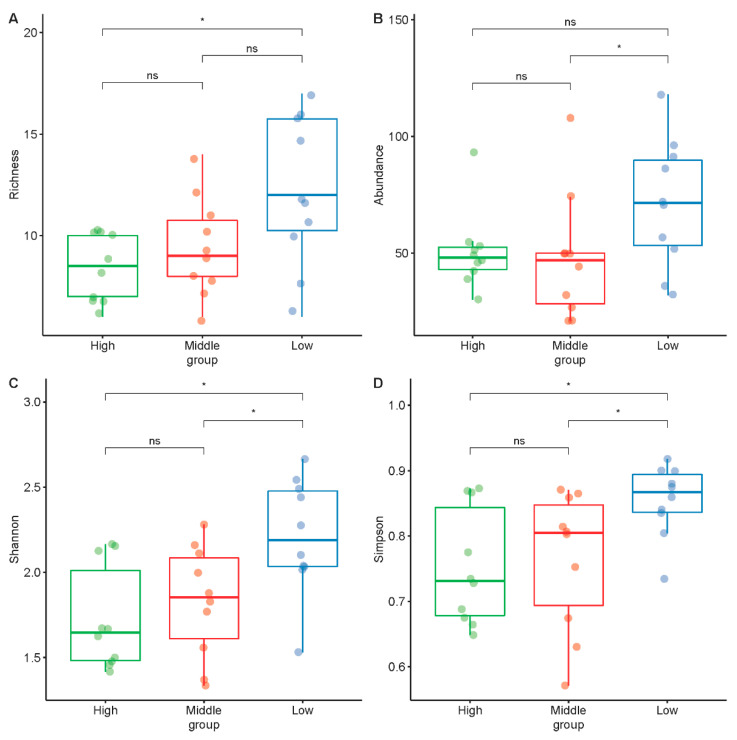
Differences in bird richness (**A**), abundance (**B**), Shannon diversity (**C**) and Simpson diversity (**D**) vary along three disturbances of urban mountain parks in Fuzhou, China. Point counts and questionnaires were delivered in three disturbance levels urban mountain parks in Fuzhou, China. Star notation indicates the significance level of analysis with Wilcoxon rank-sum tests (ns = not significant; * = *p* < 0.05).

In the figure of richness, there was a significant difference between low disturbance and high disturbance. Additionally, the result also showed that low disturbance had higher species richness and abundance. Additionally, in terms of the two gradients of moderate and high, their species composition and abundance were relatively similar. However, there was a significant difference in the abundance between low and moderate disturbances. In terms of Shannon diversity and Simpson diversity, the low disturbance was significantly higher than the other two disturbances (see Figure 5).

**Figure 6:**
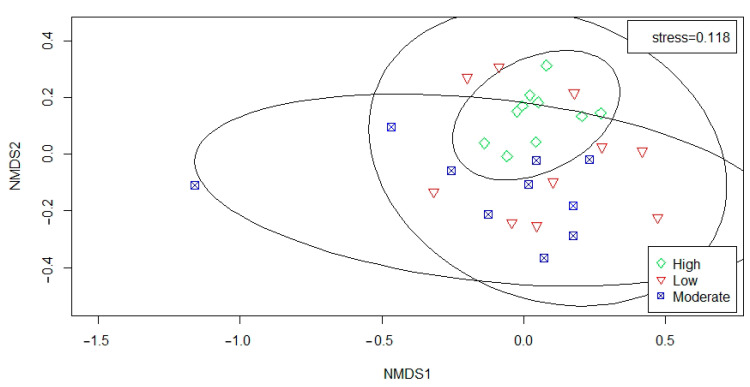
NMDS two-dimensional plot of bird assemblages from 30 sampled sites. The ellipse indicates the 95% confidence interval for each disturbance level.

After performing 999 permutations on the bird data collected in the three disturbance levels of mountain parks, according to Figure 6, the stress value was 0.118, indicating that the results displayed by NMDS had a suitable degree of fit. The spots represented sample sites where we conducted the bird survey and different colors and shapes represented additional sample grouping information. It can be seen from Figure 6 that the repetitiveness of bird communities of moderate and high disturbance levels was more potent, and low disturbance levels were weaker. Additionally, the result of the analysis of variance showed significant differences in the bird communities along with the disturbance levels (ANOSIM: R = 0.109, *p* = 0.02).

### 3.3. Regression Analysis between Bird Diversity and Momentary Mental Health

After preliminary inspection and analysis, we matched the bird diversity data collected at the sample points with momentary mental health and performed linear regression models in R. The VIF values of all models are less than 10, indicating there is no potential multicollinearity. The estimate values under different disturbance levels differ significantly and present different influence mechanisms. The regression model results showed that the three emotions (positive, negative, and anxiety) were not found to be significantly related to bird diversity at all disturbance levels, as well as in three disturbance levels (see Table 2). Our results indicated no associations between momentary psychological well-being measures and bird diversity in the linear regression model.

## 4. Discussion

On a global scale, although China’s urbanization process continues rapidly, its policies for enriching the biodiversity system and conservation are also continuously advancing. However, the role of urban biodiversity in providing human well-being and ecosystem services has not yet been understood fully [56]. Here, we provide a study on the relationship between bird diversity and momentary mental health in urban mountain parks in the southeastern coastal region of China.

### 4.1. Relationship between Bird Diversity and Mental Health

Our results showed no statistically significant relationship between bird diversity and momentary mental health (positive, negative, and anxious emotions), neither on the overall disturbance gradient nor on the different levels of disturbance gradient, which is similar to Cracknell et al. (2016) [57], but different from the results of Cox et al. (2017) [41]. Nevertheless, our results were contrary to the study from Antrop (2004) [4], which concluded that the abundance was positively linked to human well-being. Additionally, a survey from Luck et al. (2011) [34] indicated that greater abundance of birds could reduce depression, anxiety, and stress and bring better life satisfaction.

The bird abundance and species richness of low-disturbance urban mountain parks were higher than moderate-disturbance and high-disturbance areas, consistent with Otieno et al. (2021) [43]. Bird species are susceptible to environmental changes, especially when disturbed by human beings, noise, the presence of predators, etc. Such a phenomenon may be that high-disturbance areas (Wushan park and Yusan park) have more visitor flows and intensified construction, leading to low bird diversity in such regions, and previous studies also confirmed similar conclusions [58,59,60]. Our results were also contrary to the “intermediate” disturbance hypothesis [61], which means we found higher bird abundance and species richness at low disturbance. The reason may be that the study urban mountain parks we selected are the main construction area of Fuzhou City, and we did not choose rural or suburban as study sites to compare with urban areas, which is also one of the focuses of future research. There are differences in the actual existence and perception of biodiversity among people from different backgrounds, indicated by Fuller et al. (2007) [26]. Additionally, the public, who are significantly less familiar with biodiversity, will misestimate actual species diversity, and it has little impact on mental health, which is consistent with our results. However, Dallimer et al. (2012) [23] demonstrated that an awareness of greater species diversity was positively associated with mental health; whether such viewpoints were equally applicable in the southeastern coastal areas of China is also worth researching in the future. The relationship between bird diversity and human well-being is complicated, not only because of the different geographical locations in the world and the use of different biodiversity scalar indicators, but also the selection of mental health indicators and the content of the settings (such as in different places or different backgrounds). Therefore, drawing a general and widespread understanding of the link between biodiversity and human well-being is still very obstructive, but it can be compensated by using similar methods [44].

### 4.2. Factors May Affect Positive, Negative, or Anxious Emotions

We found higher positive emotions and lower negative emotions and anxiety in low-disturbance mountain parks, and the negativity and anxiety in the low-disturbance parks were significantly different from the other two disturbances. Our study indicated no link between bird diversity and mental health at all levels, and we hypothesized that other factors might drive positive emotions, negative emotions, or anxiety [57].

Clucas et al. (2015) [62] demonstrated that the public’s experience of well-being may be associated with particular species encountered at the time or present in some places. For example, when encountering songbirds or other species with pleasant sounds, people often experience positive emotions and reduce negative or anxious effects. When hearing or experiencing some harsh noises or wild animals considered dangerous (such as the roar of beasts), negative emotions such as fear or wanting to escape from the place occur [63,64,65]. The low disturbance often accompanied by less urban noise and reasonable development makes such areas a harmonious coexistence of humans and animals, which explains why low-disturbance areas have higher positive and lower negative emotions. Therefore, adequate research is needed to explore what factors affect momentary mental health and the importance of these factors. Previous studies indicated that specific attributes of green spaces, such as lighting, cleanliness, and abundance of vegetation, as well as public’s perception of features, such as nature, comfort, and beauty, may affect happiness. Higher levels of negative emotions can also be explained as concerns about personal safety, which have been shown to directly increase the negative impact of the green space of urban mountain parks. Additionally, due to the high density of vegetation cover in urban mountain parks, it may lead to fear of the confined space environment and indirectly increase the negative emotions of recreationists [38].

### 4.3. Past Experiences Make the Bird Diversity Perceived Differently

There are significant differences in the biodiversity perceived among people with different past experiences, and there are also differences in their perceptions of objective reality. Ratcliffe et al. (2018) proved that the observed biodiversity differed from what people perceived [66]. It is straightforward to be misestimated by visits and has a weaker association with mental health. The public’s perceptions may be affected by their past experiences or cognition [67], affecting their positive or negative emotional reactions. For example, hearing beautiful bird songs or seeing a docile species may increase positive emotions. Mental health may be affected by specific species or environments. In the study on the well-being of biodiversity, there may be a large difference between the objectively existing species richness or abundance perceived by people [26]. Previous studies also demonstrated that bird species usually affect mental health through their attractive appearance and pleasant calls or other factors that can be perceived by the public [33,66,67]. Additionally, for people who are familiar or unfamiliar with bird species, more richness bird diversity could add more features that can be perceived by the public. In the future, the construction of urban mountain parks should focus on how to effectively build a landscape environment and attract more different bird species so that they can be easily contacted or perceived by the public. Nonetheless, it is still necessary to collect larger datasets to study the effects on people’s mental health. Moreover, the above results also emphasize that the protection of mountain parks under different construction intensities, especially the protection of high-disturbance mountain parks, needs to be taken seriously by urban construction decision makers. There is evidence that urban mountain parks can provide important habitats for endangered species. City planners should work hard to protect these spaces, protecting wild animals and human well-being.

### 4.4. Implications and Prospects

Our study found that urban mountain parks in low-disturbance areas had higher bird richness and diversity, probably because the ecological environment in low-disturbance areas is well-protected and has less human disturbance. In contrast, bird diversity in high-disturbance areas (Wushan Park and Yushan Park) remained low because of the high level if traffic, shrill urban noise, and anthropogenic disturbances (e.g., skyscrapers, concrete roads, etc.), which force plenty of bird species to migrate to habitats that are more conducive to survival. This has also been verified by previous literature [68,69,70]. Similarly, in high-disturbance areas, we have found higher levels of negative emotions and anxiety among recreationists. High-density built-up areas often accompanied by a reduction in urban green space [4]. The public may prefer more natural green spaces that provide a larger living space for biodiversity with more ecological bits to use. Therefore, we call on urban planners to build more parkland or other green areas in the environment where we live to promote the harmonious coexistence of humans, nature, and wildlife [25]. However, there are still some shortcomings in our study, and in future research on the relationship between biodiversity and human well-being, there is a need to focus on expanding the number of green space and biodiversity in multiple dimensions survey and exploring the interaction between more background people and wild plants and animals [5,9]. In addition, physiological instruments (wearable physiological instruments, electroencephalogram detectors, etc.) can be introduced to examine the public’s reactions and corresponding emotional changes in the face of biodiversity in different environments [10,29,39].

## 5. Conclusions

With the intensification of urbanization, city builders desire to pursue sustainable development and a vision of harmonious economic, social, and environmental needs. To meet these multiple needs, interdisciplinary research is essential to emphasize land-use planning interventions to obtain expected benefits. However, our study found no significant relationship between bird diversity and mental health in other disturbances of urban mountain parks, which need to be further explored in the future. Nevertheless, we recommend protecting the bird diversity of mountain parks and encouraging the public to visit different green spaces, which is significant for the harmonious coexistence of humans and creatures. Our study is essential for urban planning decision makers, natural resource protectors, and public health professionals who seek to manage the urban environment to protect wildlife while improving the quality of life of people in rapidly developing cities.

## Figures and Tables

**Table 1:** Mental health scales used in a short face-to-face questionnaire: (**a**) Positive and Negative Affect Schedule (PANAS), containing 10 positive and 10 negative words, and (**b**) State-Trait Anxiety Inventory (STAI) six-item short-form, containing six words that relate to anxiety.

(a) Positive and Negative Affect Schedule (PANAS)			
Please rate how you feel now, in this spot where you are standing. Response options: Not at all, A little, Moderately, Quite a bit, Extremely			
1. Enthusiastic	6. Distressed	11. Alert	16. Nervous
2. Active	7. Inspired	12. Upset	17. Ashamed
3. Interested	8. Strong	13. Afraid	18. Guilty
4. Determined	9. Proud	14. Scared	19. Irritable
5. Excited	10. Attentive	15. Uneasy	20. Hostile
**(b) Spielberger State-Trait Anxiety Inventory (STAI) Six-Item Short-Form:**			
Please rate how you feel now, in this spot where you are standing. Response options: Not at all, A little, Moderately, Quite a bit, Extremely			
1. Calm 2. Tense 3. Upset 4. Relaxed 5. Content 6. Worried			

**Table 2:** Estimate for linear regression models testing whether three measures of psychological well-being (positive affect, negative affect, and anxiety) can be predicted by four different measures of bird diversity (species richness, abundance, Shannon diversity, and Simpson diversity) across all sites, high disturbance sites, moderate disturbance sites, and low disturbance sites. Star notation indicates significance level.

Disturbance Levels	Bird Diversity	Estimate		
		Positive	Negative	Anxiety
All disturbance levels (*n* = 30)	Shannon	4.103	−6.414	−5.904
	Simpson	16.100	−20.550	−18.78
	Abundance	0.025	−0.044	−0.157
	Richness	0.407	−0.730	−0.901
High disturbance (*n* = 10)	Shannon	−4.051	−4.323	14.320
	Simpson	−10.08	−8.751	26.94
	Abundance	0.120	−0.011	−0.193
	Richness	−0.423	−1.103	3.999
Moderate disturbance (*n* = 10)	Shannon	6.337	1.469	3.564
	Simpson	22.570	4.951	17.430
	Abundance	−0.085	0.028	−0.088
	Richness	0.048	0.164	−0.412
Low disturbance (*n* = 10)	Shannon	−0.520	0.220	0.254
	Simpson	−8.530	2.381	6.337
	Abundance	0.040	0.021	−0.003
	Richness	−0.021	0.054	−0.041

## Data Availability

The data used to support the findings of this study are available from the corresponding author upon request.
